# Immunomodulatory Responses of Two Synthetic Peptides against *Salmonella* Typhimurium Infection

**DOI:** 10.3390/molecules26185573

**Published:** 2021-09-14

**Authors:** Marco Antonio Ibarra-Valencia, Gerardo Pável Espino-Solis, Blanca Elisa Estrada, Gerardo Corzo

**Affiliations:** 1 Departamento de Medicina Molecular y Bioprocesos, Instituto de Biotecnología, Universidad Nacional Autónoma de México, A.P. 510-3, Cuernavaca 62250, Mexico; 2 Laboratorio de Investigación Traslacional and Laboratorio Nacional de Citometría de Flujo-UACH, Universidad Autónoma de Chihuahua, Circuito Universitario, Campus II, Chihuahua 31109, Mexico; gespinos@uach.mx (G.P.E.-S.); P309358@uach.mx (B.E.E.)

**Keywords:** antimicrobial peptides, *Salmonella enterica* serovar Typhimurium, cytokines, inflammation, scorpion peptides

## Abstract

In vitro assays of phagocytic activity showed that the peptide Pin2[G] stimulates phagocytosis in BMDM cells from 0.15 to 1.25 μg/mL, and in RAW 264.7 cells at 0.31 μg/mL. In the same way, the peptide FA1 induced phagocytosis in BMDM cells from 1.17 to 4.69 μg/mL and in RAW 264.7 cells at 150 μg/mL. Cytokine profiles of uninfected RAW 264.7 showed that Pin2[G] increased liberation TNF (from 1.25 to 10 μg/mL) and MCP-1 (10 μg/mL), and FA1 also increased the release of TNF (from 18.75 to 75 μg/mL) but did not increase the liberation of MCP-1. In RAW 264.7 macrophages infected with *Salmonella enterica* serovar Typhimurium, the expression of TNF increases with Pin2[G] (1.25–10 μg/mL) or FA1 (18.75–75 μg/mL). In these cells, FA1 also increases the expression of IL-12p70, IL-10 and IFN-γ when applied at concentrations of 37.5, 75 and 150 μg/mL, respectively. On the other hand, stimulation with 1.25 and 10 μg/mL of Pin2[G] promotes the expression of MCP-1 and IL-12p70, respectively. Finally, peptides treatment did not resolve murine gastric infection, but improves their physical condition. Cytokine profiles showed that FA1 reduces IFN-γ and MCP-1 but increases IL-10, while Pin2[G] reduces IFN-γ but increases the liberation of IL-6 and IL-12p70. This data suggests a promising activity of FA1 and Pin2[G] as immunomodulators of gastric infections in *S.* Typhimurium.

## 1. Introduction

The species belonging to the genus *Salmonella* are of particular interest given their high degree of dissemination in the population. Actually, their pathogenicity and resistance to antibiotics makes them part of the group of emergency microorganisms for the development of new antibiotics [1]. *Salmonella enterica* is a facultative intracellular, Gram-negative, flagellated bacillus that causes gastrointestinal diseases in humans and animals. According to its serological characteristics, *S. enterica* is classified into several serovars, the most important are *S*. *enterica* serovar Typhi and *S. enterica* serovar Typhimurium. It is estimated that *S.* Typhi infects 10.9 million people worldwide annually and causes approximately 116,800 deaths related to typhoid fever in the same period of time [2,3,4]. Meanwhile, *S*. Typhimurium, and others species of non-typhoid *Salmonella* (NTS), causes 94 million infections worldwide [4].

In humans, *S.* Typhimurium induces a self-limiting gastrointestinal infection that differs from typhoid fever caused by *S*. Typhi, however, mice infected with *S*. Typhimurium present a disease similar to human typhoid fever, therefore they are an appropriate study model for understanding this disease and developing therapies to alleviate it [4].

*S*. Typhimurium is acquired orally mainly by contaminated food and binds to epithelial cells of the small intestine through the expression of adhesins and fimbriae. Furthermore, *Salmonella* uses virulence factors to cross the epithelial barrier, which can be by crossing the M cells of Peyer’s patches, promoting their internalization by non-phagocytic enterocytes, disrupting the adherent unions of the epithelium or being phagocytosed by immune cells such as macrophages. Once *Salmonella* lies within cytoplasmic vacuoles, it starts its replicating process and spreads through mesenteric lymph nodes to colonize organs like the liver and spleen. Intestinal damage and inflammation causes the recruitment of neutrophils and the release of pro-inflammatory cytokines, such as: IL-8, IL-10, IL-1β and IL-23, among others [5,6]. This, added to the appearance of multi-drug resistant strains, has led to the need to generate new antibacterial agents that are not very prone to the development of resistance and that consider the host’s immune response as part of their mechanism of action [7,8].

A proposal for drugs that meet these conditions are the antimicrobial peptides (AMP) recently redesigned as host defense peptides (HDP). These peptides are generally short cationic amino acid chains, amphipathic and widely distributed in nature. They can prevail as alpha helices, beta chains, random structures or as a combination of these three structures [9]. As mentioned previously, HDP are present in a wide variety of living organisms, such as plants, animals and insects.

Particularly, scorpion venom is an important source of medicinal molecules and several HDP [10]. Taking this into consideration, the synthetic antimicrobial peptides Pin2[G] and FA1 were developed, the first is a derivative of pandinin 2 from *Pandinus imperator* [11,12] and the second one is a chimera of two HDPs, the region N-terminal of vejovine and the C-terminal of hadrurin, from *Vejovis mexicanus* and *Hadrurus gertschi*, respectively [13]. Previously, its ability to counteract infections against reference strains and clinical isolates of *Staphylococcus aureus* and *Pseudomonas aeruginosa* was reported at in vitro level and in in vivo models of skin infection [14]. At the same time, it was observed that both peptides have certain effects on the release of MCP-1 and IL-6 by RAW 264.7 and HBE macrophages [14]. Furthermore, in a model of gastrointestinal infection by *S*. Typhimurium ATCC 14028, it was observed that the administration of Pin2[G] intravenously reduces bacterial presence in feces, but does not completely inhibit it. Therefore, our interest arises to understand the role of such peptides as immunomodulators in this type of infections.

Here we show that both peptides promote phagocytosis of *S.* Typhimurium by macrophages and modulate the release of inflammatory cytokines under basal and infection conditions. Although intestinal infection in murine models was not resolved, there was a decrease in the number of CFUs isolated from feces and some protection towards infection behavior accompanied by reduced cytokine levels in plasma. Thus, Pin2[G] and FA1 could be considered as leads for developing therapeutic peptides against intestinal infections by *S.* Typhimurium.

## 2. Results

### 2.1. Pin2[G] and FA1 Are Innocuous to Viability of Macrophages

**Figure 1:**
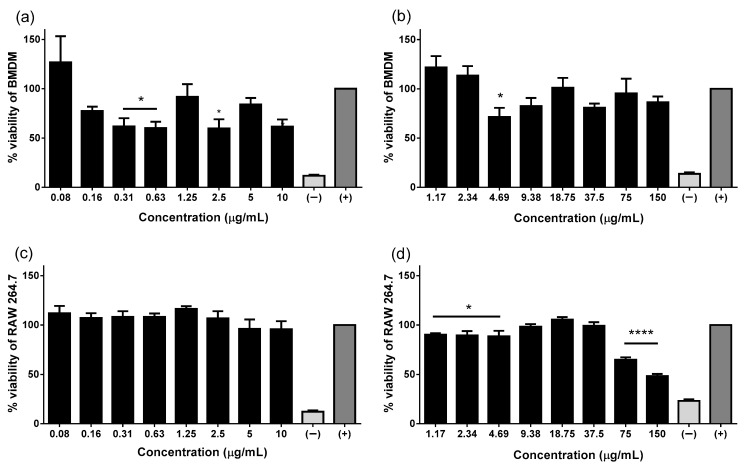
Pin2[G] and FA1 do not alter viability of murine macrophages. Percentage of viability of bone marrow-derived macrophages (BMDM) and RAW 264.7 cells stimulated with Pin2[G] (**a**,**c**) and FA1 (**b**,**d**) was evaluated. In both cases 1 × 10^5^ cells were incubated overnight with different concentrations of peptides. Cells without stimulus were used as positive control (+) and cells lysed with Triton X-100 served as negative control (−). The mean ± standard deviation of at least three independent experiments is shown. Asterisks denote significant differences between treatments and negative control. *p* = * <0.05, **** <0.0001.

Since one of the drawbacks of some HDPs, including Pin2[G] and FA1, is their toxic effect on eukaryotic cells, Pin2[G] and FA1peptide concentrations used for this work, without jeopardizing macrophages viability, were tested based on previous reports [14]. Thus, RAW 264.7 and BMDM were cultured in the presence of different peptides concentrations for 24 h and cell viability was evaluated. Results suggest that Pin2[G] decreases cell viability of BMDM by almost half at 0.16, 0.31, 0.63, 2.5 and 10 µg/mL (Figure 1a). Likewise, FA1 also diminishes cell population but not at a significant level, except at 4.69 µg/mL (Figure 1b). On the other hand, in RAW 264.7 cells, FA1 reduced cell populations at 75 and 150 µg/mL and slightly at 1.17–4.69 µg/mL while Pin2[G] does not affect these types of cells (Figure 1d).

### 2.2. Both Peptides Stimulate Phagocytosis of S. Typhimurium by Macrophages

**Figure 2:**
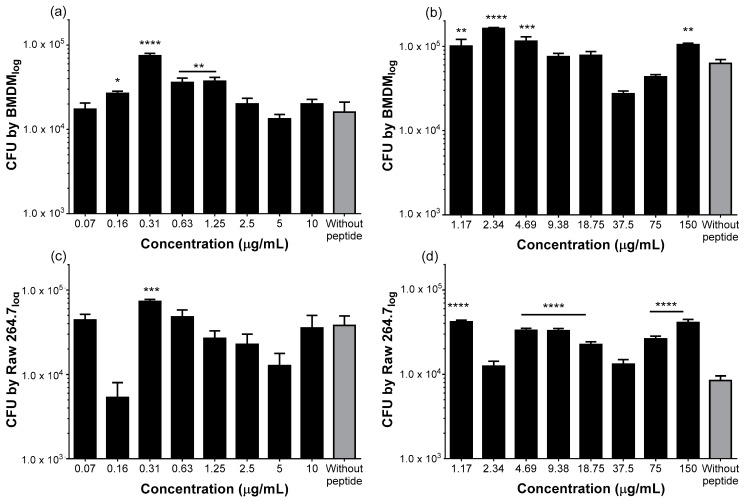
Pin2[G] and FA1 promote phagocytosis of *S.* Typhimurium by murine macrophages. Macrophages at a density of 1 × 10^5^ cells/well were challenged with different concentrations of Pin2[G] (**a**,**c**) and FA1 (**b**,**d**) and it was determined if this affects the macrophage ability to engulf 10^6^ CFU *S.* Typhimurium/well. The mean ± standard deviation of at least three independent experiments is shown. Asterisks denote significant differences between treatments and negative control, cells without peptide. *p* = * <0.05, ** <0.005, *** <0.0005, **** <0.0001.

One activity that has been attributed to HDPs is their influence on the activity of immune cells, such as their phagocytic capacity. Therefore, in order to determine if Pin2[G] and FA1 were able to stimulate macrophages to engulf bacteria, BMDM (Figure 2a,b) and RAW 264.7 (Figure 2c,d) cells were challenged for 24 h with the same peptide concentrations as in the previous viability assay, and then incubated with *Salmonella* Typhimurium for 30 min. Cells were later lysed and the number of phagocytized bacteria was counted. Figure 2a shows that there was an increase in *S.* Typhimurium phagocytosis, with respect to the non-stimulated cells (control) when BMDM were incubated with 0.16–1.25 µg/mL of Pin2[G] with a maximum at 0.31 µg/mL. Using the same model, FA1 stimulates phagocytosis at concentrations from 1.17 to 4.69 µg/mL with a maximum at 2.34 µg/mL, nonetheless it also showed an inhibitory effect at 37.5 µg/mL (Figure 2b). Finally, in RAW 264.7 cells, Pin2[G] slightly increases the phagocytic effect at 0.31 µg/mL (Figure 2c), while FA1 stimulates phagocytosis at all the concentrations except at 2.34 and 37.5 µg/mL (Figure 2d). Thus, both peptides Pin2[G] and FA1 influence the phagocytic activity of RAW 264.7 and BMDM macrophages.

### 2.3. Pin2[G] and FA1 Influence the Cytokine Profiles of Macrophages Infected with S. Typhimurium

**Figure 3:**
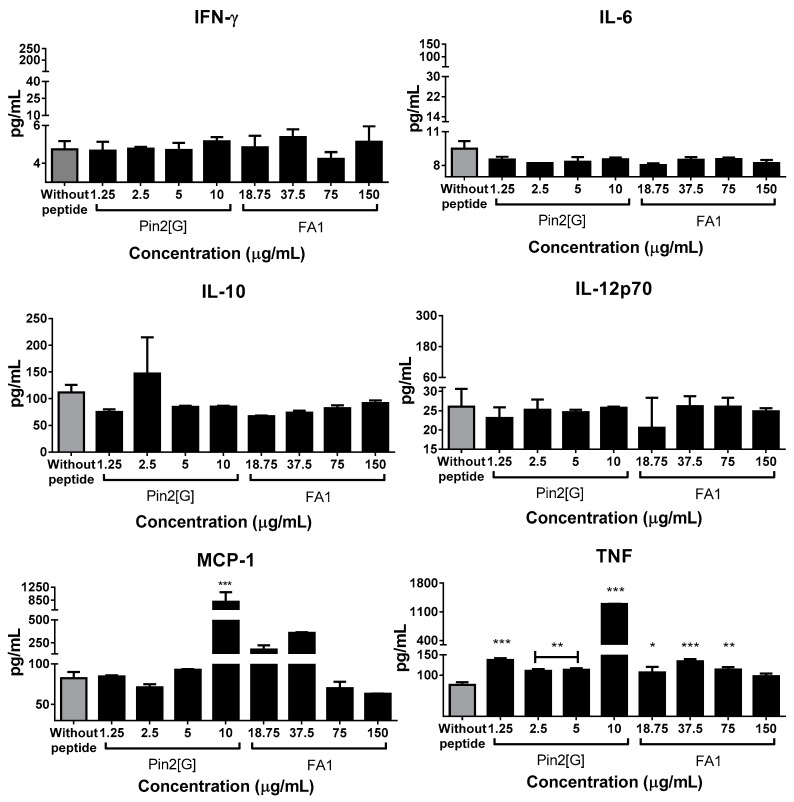
Peptides modify MCP-1 and TNF expression in unstimulated mouse macrophages RAW 264.7. Cytokine profile of the macrophages used in the phagocytosis stimulation assays prior to infection was analyzed. This was done by BD cytometric bead array (CBA) mouse inflammation kit using an Attune NxT Acoustic flow cytometer, by acquiring 10,000 events at a rate of 100 µL/min. The mean ± standard deviation of at least three independent experiments is shown. Asterisks denote significant differences between treatments and negative control (cells without peptide). *p* = * <0.05, ** <0.005, *** <0.0005.

**Figure 4:**
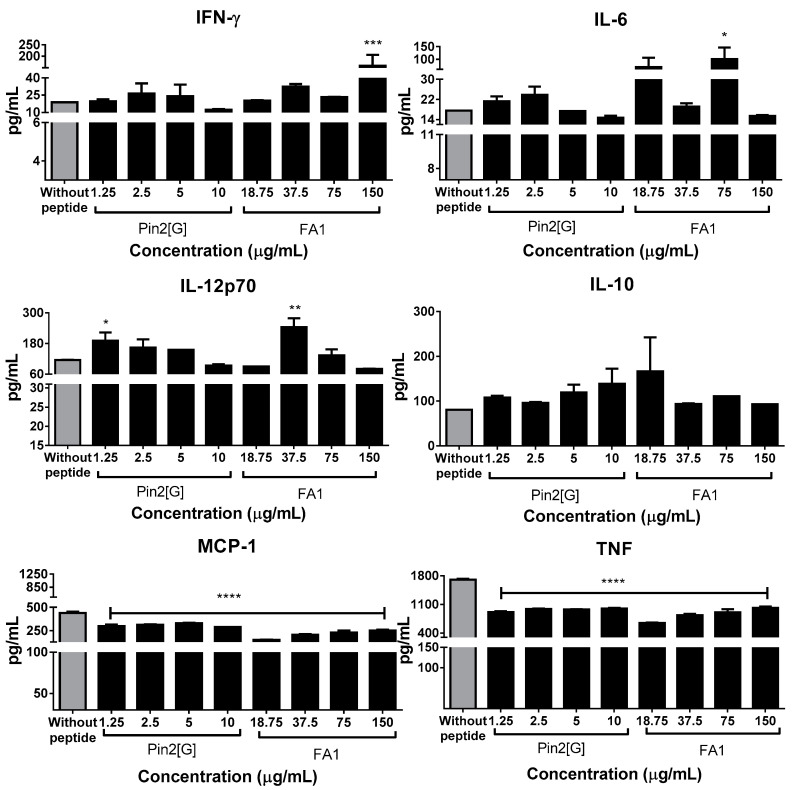
Pin2[G] and FA1 modifies cytokine expression profile in macrophages infected with *S.* Typhimurium. Cytokine profile of macrophages from phagocytosis assays was analyzed. This through flow cytometry using an Attune NxT Acoustic flow cytometer with the BD cytometric bead array (CBA) mouse inflammation kit. Furthermore, 10,000 events were recorded at a rate of 100 µL/min. The mean ± standard deviation of at least three independent experiments is shown. Asterisks denote significant differences between treatments and negative control (without peptide). *p* = * <0.05, ** <0.005, *** <0.0005, **** <0.0001.

An important aspect in the control of *S.* Typhimurium infection is the modulation of the immune response; thus, various reports have demonstrated the effect of HDP on cytokine expression profiles. Thus, by using a cytometric bead array (CBA) multiplex strategy through flow cytometry, the cytokine expression profile of RAW 264.7 cells during phagocytosis stimulation experiments was analyzed. That is, the cytokines present in the macrophage’s supernatant after 24 h incubation with different concentrations of peptides, followed by challenge with *S.* Typhimurium 30 min later were evaluated (Figure 3 and Figure 4). It was observed that in basal conditions, Pin2[G] stimulates MCP-1 at 10 µg/mL. Likewise, Pin2[G] from 1.25 to 10 µg/mL, and FA1 from 18.75 to 75 µg/mL increased the liberation of TNF (Figure 3).

In contrast to this, cells infected with *S.* Typhimurium showed a rise of IFN-γ and Il-6 with 75 and 150 µg/mL of FA1, respectively. Regarding IL-12p70, its expression increases with 1.25 and 37.5 µg/mL of Pin2[G] and FA1, respectively. Finally, Pin2[G] from 1.25 to 10 µg/mL and FA1 from 18.75 to 150 µg/mL, decreases MCP-1, the same effect of using the same concentrations of both peptides is observed in the TNF profile (Figure 4). In summary, Pin2[G] and FA1 modulate cytokine profiles of macrophages according to the context of the cells.

### 2.4. Both HDP Decreases Salmonella Typhimurium Gastrointestinal Infection

**Figure 5:**
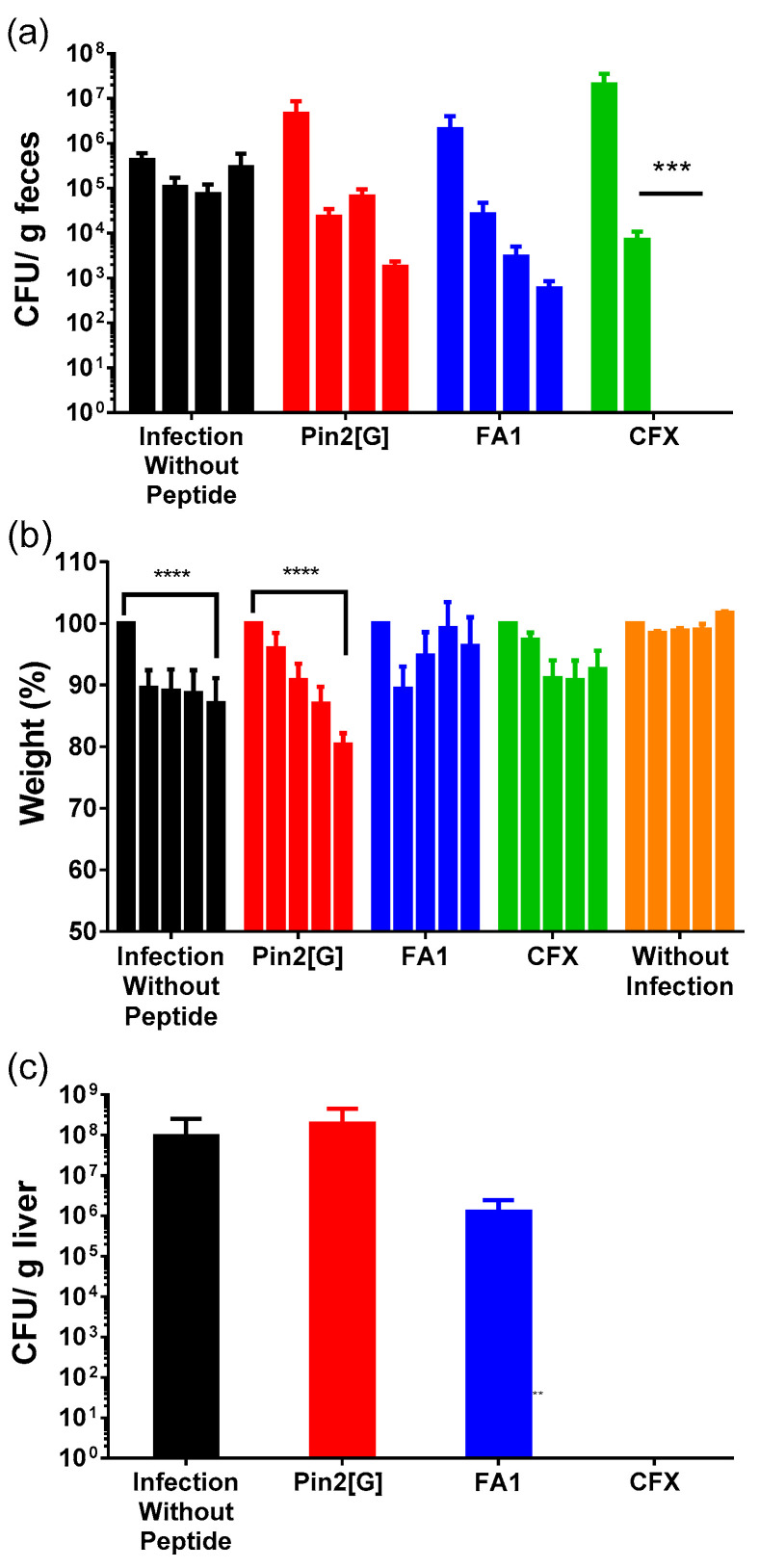
Pin2[G] and FA1 decreases *S.* Typhimurium gastrointestinal infection. (**a**) Bacterial count in stools. For four days, the number of bacteria present in feces was counted every 24 h by serial dilution to assess the progress of infection. (**b**) Evolution of mice weight during infection. For four days, the mice were weighed every 24 h to record the effect of the infection and of the treatment on their physical state. (**c**) Bacterial count in liver. At the end of the treatment mice were sacrificed and their livers were removed, macerated to isolate and quantify the bacterial load present in it by serial dilution. Infection clusters without peptide are shown; infected treated with FA1 (5 mg/kg); infected treated with Pin2[G] (2.5 mg/kg); infected treated with CFX (70 mg/kg) and a group without infection. The mean ± standard deviation, of three independent experiments with three individuals in each one, is shown. Asterisks denote significant differences between treatments and negative control (infected mice without treatment). *p* = *** <0.0005, **** <0.0001.

Finally, the ultimate importance of the development of new antimicrobials lies in their application in vivo in order to understand the function of peptides in complex organisms. Thus, the antimicrobial effect of Pin2[G] and FA1 was observed in a model of gastrointestinal infection by *S.* Typhimurium ATCC14028 in BALB/c mice. Pin2[G] (2.5 mg/kg), FA1 (5 mg/kg) and ceftriaxone (70 mg/kg) were used as treatment, each group had three individuals and it was repeated on three different occasions. The dose of Pin2[G] and FA1 corresponds to the range of concentrations that can be administered to mice without being toxic to them, while the dose of ceftriaxone (CFX) refers to that recommended by health agencies for the treatment of *Salmonella* Typhimurium [14,15,16]. Likewise, an independent group of infected mice lacking peptide treatment was considered as a control (Figure 5)**.** Mice treated with Pin2[G] and FA1 reduced bacterial load in feces with respect to the onset of infection, and the control group; however, the infection was not eradicated as it was in the group that received the antibiotic ceftriaxone (Figure 5a). Regarding the variation in weight, mice that received FA1 managed to regain weight from the second day, while groups that received Pin2[G] and without treatment lost weight till the end of the experiment. It should be mentioned that treatment with ceftriaxone did not favor recovery or weight, as FA1 did (Figure 5b). Finally, none of the synthetic peptides prevented colonization in the liver (Figure 5c).

It is worth mentioning that during the time of experimentation, mice that received FA1 maintained healthy behavior; that is, no bristling hair, stooping or morphological alteration of the stool were observed. With this, it can be concluded that no single peptide, Pin2[G] or FA1, can inhibit the *S.* Typhimurium infectious process, but they can reduce the number of infecting bacteria and loss of body mass.

### 2.5. Pin2[G] Promotes Inflammatory Cytokines While FA1 Stimulates an Anti-Inflammatory Profile

**Figure 6:**
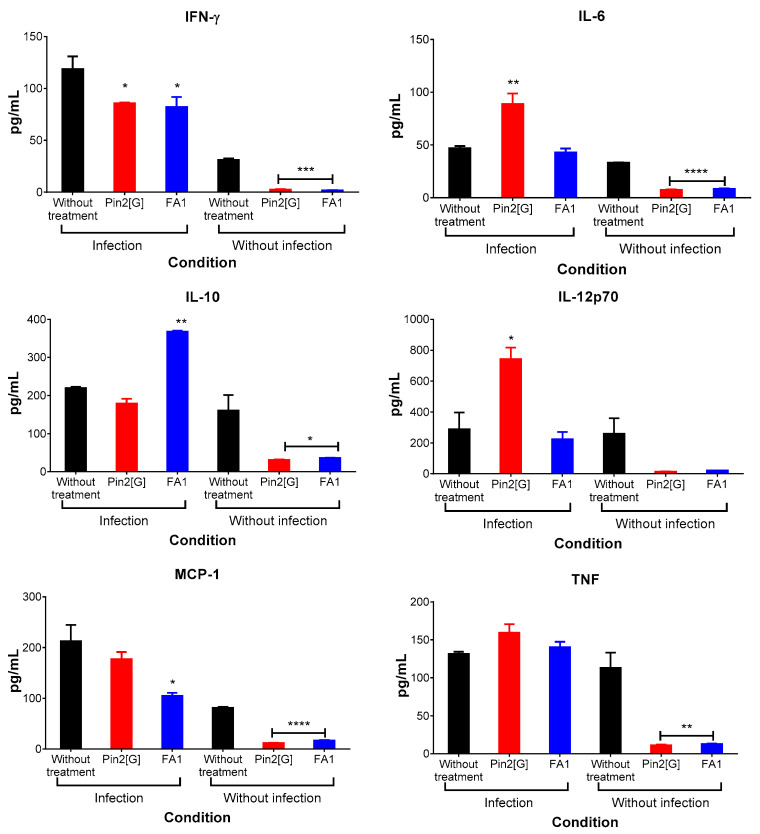
Pin2[G] promotes inflammatory cytokine expression profile in mouse plasma while FA1 promotes an anti-inflammatory profile. Cytokine profile was analyzed by flow cytometry using an Attune NxT Acoustic flow cytometer with the BD cytometric bead array (CBA) mouse inflammation kit. Furthermore, 10,000 events were recorded at a rate of 100 µL/min. Mean ± standard deviation of two independent experiments is shown. Asterisks denote significant differences between treatments and mice infected without treatment. *p* = * <0.05, ** <0.005, *** <0.0005, **** <0.0001.

As mentioned before, an important part of *S.* Typhimurium infection is the host immune response, so it is important to determine if our peptides have an effect on it. This was analyzed, as in the in vitro models, using the determination of cytokine profiles. According to this, after treatment with Pin2[G] and FA1, the expression profile of cytokines IL-6, IFN-γ, TNF, IL-10, IL-12p70 and MCP1 in plasma was determined (Figure 6). It was observed that Pin2[G] has an inflammatory effect by increasing the expression of IL-6 and IL-12p70 and even exceeds the group of mice infected with *S*. Typhimurium. However, it also decreases the levels of IFN-γ. In contrast, FA1 diminishes the expression of IFN-γ and MCP-1 but increases the concentration of IL-10. As can be observed, Pin2[G] promotes the release of inflammatory cytokines, while FA1 promotes anti-inflammatory response.

## 3. Discussion

Understanding antimicrobial and immunological characteristics of AMPs could perhaps place them at the forefront in the fight against diseases caused by multidrug resistant bacteria [17]. One factor to consider is that, during the resolution of an infection, antimicrobial and immunomodulatory qualities could act together. Even a peptide that lacks antibacterial effect can eliminate a bacterial infection due to its effect on the immune system by modulating the release of inflammatory molecules or by activating cellular events such as chemotaxis, phagocytosis, proliferation or cellular polarization [18,19].

Although particular effort has been made to implement therapies based on the use and stimulation of the release of natural human AMP, like cathelicidins and beta-defensins, the relevance and variety of antimicrobial peptides that can be obtained from lower animals such as venomous spiders, scorpions, snakes and frogs is undeniable [20]. In previous studies, the antimicrobial effect of the synthetic peptides Pin2[G] and FA1 was demonstrated against multidrug-resistant clinical isolates of *Staphylococcus aureus* and *Pseudomonas aeruginosa* in topical infection models, as well as their effect against erythrocytes and leukocyte cell lines [14]. Furthermore, their stability in various physiological fluids and culture media was observed [21]. This led us to consider them as viable agents in the treatment of bacterial infections.

Here, we tested the potential of Pin2[G] and FA1 as antimicrobial peptides and host defense peptides, against gastrointestinal infection by *Salmonella* Typhimurium. This, considering their immunological potential as modulators of inflammation by cytokines and surveillance at in vitro and in vivo level. At in vitro level, it was observed that both peptides have certain effects on the viability of bone marrow-derived macrophages (BMDM) and the RAW 264.7 cell line by decreasing it at different concentrations. This decrease is not representative, in the case of FA1, but it is in that of Pin2[G]. Thus, the range of use of Pin2[G] turns out to be more limited, which should be considered to improve treatment at the systemic level. An interesting observation is that macrophage toxicity was not a concentration-dependent event, which is the classic mechanism of action of AMPs. Therefore, some other biochemical mechanisms may be hampered at low concentrations of such peptides; for instance, peptide interaction with some cell targets that affect viability, or even interference with DNA replication. This effect of subinhibitory concentrations of peptides has previously been reported [22,23]. However, a more detailed explanation requires collecting more information related to the interaction of Pin2[G] and FA1 with proteins or genetic elements of immune cells.

Likewise, it is interesting to note that Pin2[G] stimulates phagocytosis of *S.* Typhimurium by BMDM at low concentrations (0.31–1.25 µg/mL) and with 0.31 µg/mL on RAW 264.7 cells, an effect that was also achieved with FA1 at a concentration range from 1.17 to 4.69 µg/mL in BMDM. While in RAW 264.7 cells, this effect occurs by FA1 at 1.17 and from 4.69–18.75 µg/mL. FA1 also decreased the phagocytic action of both cell types at 37.5 µg/mL, which refers to a range of activity also present in other peptides [24].

Regarding the cytokine expression profiles in RAW 264.7 cells, it was observed that Pin2[G] at 10 µg/mL increases the release of MCP-1. At the same time, TNF liberation was stimulated from 1.25 to 10 µg/mL of Pin2[G] and from 18.75 to 75 µg/mL of FA1.

On the other hand, cells challenged with peptides and subsequently infected with *Salmonella* Typhimurium presented a different profile, first, both peptides decrease MCP-1 and TNF levels, but the main differences lie in IL-12p70, IL-10 and IFN-γ, which were stimulated by FA1 at 37.5, 75 and 150 μg/mL, respectively, while Pin2[G] rises the expression of IL-12p70 with 1.25 μg/mL. The mentioned cytokines are usually among the most studied, because they are the most relevant in terms of activating several immune processes. This is why it is known that peptides such as KT2, RT2, cathelicidin LL-37 and defensins HBD2 and HBD3 also have an inhibitory effect on the release of inflammatory cytokines [18,25,26].

Although both types of macrophages belong to the same cell lineage, their response to this two HDPs was particular of each one. This might be related to being a commercial lineage, in the case of RAW 264.7, or a wild type model, talking about BMDM, which adds evidence to the importance in the choice of the biological model during the development of experiments that involves cellular response to external stimulus.

With reference to the in vivo model of gastrointestinal infection by *Salmonella* Typhimurium, it was observed that treatment with Pin2[G] (2.5 mg/kg) reduces the bacterial load and promotes inflammation by stimulating the release of IL-6 and IL-12p70, but diminishing the release of IFN-γ; thus far it is unknown if these effects are related between them. On the other hand, FA1 (5 mg/kg) decreases bacterial load without inhibiting it, in addition to decreasing the expression of IFN-γ, IL-6 and MCP-1 at the time increasing the release of IL-10 in plasma, thus presenting an anti-inflammatory effect. This could be associated with the conservation of the physical state of the mice, which was reflected by their constant weight during the time that the treatment lasted. Here, we would like to highlight how Pin2[G] has an anti-inflammatory profile in vitro and inflammatory in vivo, while FA1 acts as an anti-inflammatory in both cases.

Although both peptides do not completely eliminate the infection, they do decrease bacterial load in feces, at the same time modulating the immune response. This effect has been seen in other infection models that were also treated with antimicrobial peptides [27,28,29,30]. Therefore, the possibility that a modification in the dosage, or its use in conjunction with antibiotics, will allow development of better conditions for treatment that takes into account both microbiological and immunological aspects. We are currently working on the design of treatments based on the joint administration of FA1 or Pin2[G] together with ceftriaxone. Although some interesting results have been observed at the level of cytokine expression profiles, we still need to verify this information to eventually publish it. 

As was mentioned, some antimicrobial peptides obtained from insects and arachnids have high potential as antibacterials and immunomodulators [10,20]. Such is the case of the peptide from the scorpion, *Tityus obscurus*, ToAP2, which promotes chemotaxis of peritoneal macrophages in C57BL/6 mice infected with *Mycobacterium massiliense* [31]. Furthermore, melittin, a bee peptide, causes cell death of PMBC by apoptosis [32]. Or that of cecropin AD, which protects weaned pigs from intestinal infection by *Escherichia coli* by enhancing their immune response [33]. Furthermore, the antifungal peptide TistH, from the scorpion, *Tityus stigmurus*, inhibits the growth of *Candida albicans* and *C. tropicalis* without being cytotoxic to murine leukocytes and devoid of inflammatory effects in mice [34]. Therefore, Pin2[G], a peptide derived from *Pandinus imperator* [11,20], and FA1, a chimera synthetic peptide derived from the scorpions, *Vaejovis mexicanus* and *Hadrurus gertschi* [13], are modified antimicrobials peptides of natural origin that could favor the development of novel molecules, with interesting antibacterial and immune effects.

Lastly, the door is open to deeply study the effects of AMPs/HDPs on different cells, tissues and organisms, in order to have a better understanding of their mechanism of action that in turn will lead us to the development of new therapeutic agents to counteract infectious diseases caused by multidrug-resistant bacteria.

## 4. Materials and Methods

### 4.1. Bacterial Strains and Cell Lines

*Salmonella enterica* serovar Typhimurium ATCC 14028 (*S.* Typhimurium) stored at −76 °C in 30% glycerol was used in this study. A *S.* Typhimurium striatum was performed on Xylose-Lysine deoxycholate (XLD) agar and incubated overnight at 37 °C. Isolated colonies were obtained, and one of these was inoculated in 3 mL of LB broth and incubated at 37 °C for 18 h. This process was used for the in vitro and in vivo experiments. On the other hand, the macrophage cell line RAW 264.7 from BALB/c mice was kindly donated by Drs. Mayra Silva Miranda and Silvia Andrea Moreno Mendieta from the Institute for Biomedical Investigation at the National Autonomous University of Mexico. For the viability and phagocytic activity assays, RAW 264.7 cells were grown in RPMI broth supplemented with 10% of inactivated fetal bovine serum iFBS at 37 °C with 5% CO_2_ and seeded on 96 wells plates at a density of 1 × 10^5^ cells/well.

### 4.2. Animals

For gastric infection models, BALB/C male mice weighing 19–22 g were used. They were kept in separate boxes by working group in light/dark conditions 12:12 h with food and water ad libitum. Recommendations of the Guide for the care and use of laboratory animals [35] were followed to ensure the correct use of animals. All animal procedures were approved by the bioethics committee of the Institute of Biotechnology, UNAM, based on NOM-062-ZOO-1999, project number 317 [36].

### 4.3. Viability Assay

To determine the effect of the peptides on cell integrity, CellTiter 96 AQueous reagent was used, which contains a tetrazolium (3-(4,5-dimethylthiazol-2-yl)-5-(3-carboxymethoxyphenyl)-reagent. 2-(4-sulfophenyl)-2H-tetrazolium, MTS) and an electron coupler (phenazine ethosulfate, PES). This assay consists on the reduction of MTS by living cells in a soluble formazan product in culture medium, whose absorbance can be measured at 490 nm. Formazan production is directly proportional to living cells. For this, murine macrophages RAW 264.7 or bone marrow derived macrophages (BMDM) from C57BL/6 mice were seeded in 96-well plates at a density of 1 × 10^5^ cell/well with RPMI+10%iFBS. Macrophages were exposed to different Pin2[G] (0.08, 0.16, 0.31, 0.63, 1.25, 2.5, 5 and 10 µg/mL) or FA1(1.17, 2.34, 4.69, 9.38, 18.75, 37.5, 75 and 150 µg/mL) concentrations. Such peptide concentrations were used based on a previous report; that is, Pin2 [G] and FA1 concentrations higher than 10 and 150 µg/mL, respectively, are toxic for RAW 264 cells [14]. Macrophages treated with 0.5% Triton or left without stimulation were considered as negative and positive controls, respectively. After 24-h incubation with the peptides at 37 °C in 5% CO_2_, supernatants were separated and stored at −72 °C for subsequent cytometry analysis. Fresh culture medium and 20 µL of CellTiter 96 AQueous One Solution Reagent was added and incubated for an hour at the conditions already mentioned. Absorbance at 490 nm was recorded using a plate reader. Experiments were carried out in triplicate. Results are shown as percentage of viability in regard to negative control.

### 4.4. Phagocytosis Stimulation

To observe whether macrophages stimulated with peptides increased their ability to engulf *Salmonella* Typhimurium phagocytosis stimulation assays were performed. For this, 1 × 10^5^ RAW 264.7 or BMDM were seeded in 96-well plates with RPMI medium + 10% iFBS and incubated overnight at 37 °C with 5% CO_2_. Subsequently, cell culture medium was removed and replaced with new medium RPMI supplemented with different concentrations of FA1 (1.17–150 µg/mL) or Pin2[G] (0.08–10 µg/mL) these cells were incubated at 37 °C with 5% CO_2_ for 24 h. Henceforward, supernatants were removed and frozen at −20 °C for further analysis by flow cytometry. Later, new medium with 1 × 10^6^ *S*. Typhimurium ATCC14028 was added at a 1:10 ratio, infected cells were incubated for 30 min. The culture medium was then removed and frozen at −20 °C for further analysis by flow cytometry. Next, cells were washed three times with PBS before new supplemented culture medium containing gentamicin (100 µg/mL) was added to eradicate the remaining *S*. Typhimurium cells in the wells. One hour later, the culture medium was removed and plates were washed twice with PBS. Intracellular bacteria were recovered as previously reported [37]; briefly, 100 µL of 0.05% Triton X-100 were added to each well to lyse macrophages, this suspension was centrifuged at 6000× *g* for 8 min. Supernatant was decanted and the pellet was resuspended in 1 mL of sterile PBS. Serial dilutions were then made and seeded on Xylose-Lysine deoxycholate (XLD) agar and incubated at 37 °C overnight. The number of colonies was counted and multiplied by the corresponding dilution factor to determine the number of bacteria recovered from macrophages. Each test was done in triplicate in three independent experiments. Controls of macrophages without peptide and infected with *Salmonella* Typhimurium, as a reference for basal phagocytosis without stimulus, and macrophages without peptide and without infection, to verify sterility of the culture medium were used.

### 4.5. Gastric Model Infection

Male BALB/c mice of 19–22 g were used for the infection model. Previously, we measured effects of several concentrations of Pin2[G] and FA1 on mice viability [14], this allowed us to stablish an innocuous dose for mice treatment. Hence, mice were divided into four groups (n = 3); infected mice receiving (1) PBS, (2) Pin2[G] (2.5 mg/kg), (3) FA1 (5 mg/kg) and (4) ceftriaxone (70 mg/kg). Furthermore, a group without infection as a control was included (n = 3). Data was from three independent experiments.

To induce infection, mice were sedated with chloroform, and a probe was orally introduced to administer 10^7^ CFU of *Salmonella* Typhimurium ATCC14028 in 200 µL of PBS, or just sterile PBS to uninfected mice. Each group was separated into different cages and kept in 12:12 h light/dark conditions with food and water ad libitum. Before and 24 h after infection, feces were collected from each mouse to determine the number of *S*. Typhimurium CFUs present per gram of fecal matter by serial dilutions seeded in XLD agar.

Each day, mice were weighed, behavior conditions were recorded, feces were collected to determine the number of bacteria and the treatment was administered intravenously at a volume of 200 µL. After three days of treatment, and after collecting the last stool sample, mice were sacrificed and the liver and blood were collected in sterile vials with EDTA. The liver was macerated in PBS and aliquoted to quantify bacteria. Blood was centrifuged at 1000× *g* for 5 min to separate plasma, which was frozen at −20 °C to determine cytokine profile expression.

### 4.6. Bacterial Quantification

To evaluate the evolution of the infection and the effect of the treatment, the number of CFU in feces was determined. For this, feces were collected in 2 mL Eppendorf tubes previously weighed, and after the collection, they were weighed again to calculate the weight of feces. Next, 1 mL of PBS was added to dilute the stool and from this, serial dilutions were made. Each dilution was seeded in triplicate on plates with XLD agar, which, being a differential medium, allows the identification of *S*. Typhimurium. Plates were incubated for 18 h at 37 °C and the number of colony-forming units by dilution was counted. This allowed us to determinate the number of CFUs per gram of feces (CFU/g). Each experiment was repeated three times. This same procedure was followed to determine the presence of *S*. Typhimurium in the liver.

### 4.7. Cytokine Profiles

The immunomodulatory potential of antimicrobial peptides was evaluated by determining the cytokine expression profiles. The levels of IL-6, IL-10, MCP-1, IFN-γ, IL-12p70 and TNF secreted by RAW 264.7 macrophages to the culture medium or in the plasma from mice infected with *S.* Typhimurium were analyzed by flow cytometry. For this, the BD cytometric bead array (CBA) mouse inflammation kit (Catalog No. 552364) was used following the manufacturer’s instructions. Briefly, reaction was made from a Master mix containing 5 µL of each antibody for the number of reactions to be done. Diluent solution was added to this mixture so that the volume per reaction was 50 µL of the Master mix. To this volume, 50 µL of the test sample and 25 µL of PE were added. The reaction was incubated for 2 h in the dark. Subsequently, 1 mL of wash buffer was added, and it was centrifuged for 5 min at 200× *g*. Finally, 350 µL of wash buffer was added and it was read on an Attune NxT cytometer in which 10,000 events were recorded at a flow of 100 µL/min. Results were analyzed with the FlowJo vX.0.7 program. A graph was generated comparing the size (FWD) with the complexity (SSC) of the spheres. In this way the regions of interest corresponding to the fluorescence of PE bound to the antibody were delimited and the fluorescence intensity corresponding to each cytokine was measured. With these values the concentration of each cytokine was calculated in picograms per milliliter (pg/mL). This measurement was made with two independent samples.

### 4.8. Statistical Analysis

The least significant difference method was used to determine whether statistically significant differences occurred among the mean values obtained using the software package Prism 6 (GraphPad Prism, v. 6.01, La Jolla, California, USA). For cytokine profiles and phagocytosis stimulation and viability assay, one-way analysis of variance (ANOVA) was applied to independent experiments with Fisher’s test, each done in triplicate with the GraphPad Prism 6 program. For *Salmonella* Typhimurium infection in vivo model data was analyzed with two-way ANOVA with Dunnet’s multiple comparison test. Results show the average of these experiments with standard deviation. *p* values less than 0.05 were considered statistically significant.

## Data Availability

Not applicable.

## Sample Availability

Not available.

## References

[1] World Health Organization (2014). Antimicrobial Resistance: Global Report on Surveillance.

[2] GBD 2017 Typhoid and Paratyphoid Collaborators (2019). The global burden of typhoid and paratyphoid fevers: A systematic analysis for the Global Burden of Disease Study 2017. Lancet Infect. Dis..

[3] Marchello C.S., Hong C.Y., Crump J.A. (2019). Global typhoid fever incidence: A systematic review and meta-analysis. Clin Infect Dis..

[4] Majowicz S.E., Musto J., Scallan E., Angulo F.J., Kirk M., Brien S.J.O., Jones T.F., Fazil A., Hoekstra R.M. (2010). The Global Burden of Nontyphoidal Salmonella Gastroenteritis. Clin. Infect. Dis..

[5] Broz P., Ohlson M.B., Monack D.M. (2012). Innate immune response to *Salmonella* Typhimurium, a model enteric pathogen. Gut Microbes.

[6] Hurley D., McCusker M.P., Fanning S., Martins M. (2014). *Salmonella*–Host Interactions–Modulation of the Host Innate Immune System. Front. Immunol..

[7] Boto A., De La Lastra J.M.P., González C.C. (2018). The road from host-defense peptides to a new generation of antimicrobial drugs. Molecules.

[8] Mwangi J., Hao X., Lai R., Zhang Z.Y. (2019). Antimicrobial peptides: New hope in the war against multidrug resistance. Zool. Res..

[9] Fjell C.D., Hiss J.A., Hancock R.E.W., Schneider G. (2012). Designing antimicrobial peptides: Form follows function. Nat. Rev. Drug Discov..

[10] Harrison P.L., Abdel-Rahman M.A., Miller K., Strong P.N. (2014). Antimicrobial peptides from scorpion venoms. Toxicon.

[11] Corzo G., Escoubas P., Villegas E., Barnham K.J., He W., Norton R.S., Nakajima T. (2001). Characterization of unique amphipathic antimicrobial peptides from venom of the scorpion *Pandinus imperator*. Biochem. J..

[12] Rodríguez A., Villegas E., Montoya-rosales A., Rivas-santiago B., Corzo G. (2014). Characterization of Antibacterial and Hemolytic Activity of Synthetic Pandinin 2 Variants and Their Inhibition against *Mycobacterium tuberculosis*. PLoS ONE.

[13] Sánchez-Vásquez L., Silva-Sánchez J., Jiménez-Vargas J.M., Rodríguez-Romero A., Muñoz-Garay C., Rodríguez M.C., Possani L.D. (2013). Enhanced antimicrobial activity of novel synthetic peptides derived from vejovine and hadrurin. BBA Gen. Subj..

[14] Arenas I., Ibarra M.A., Santana F.L., Villegas E., Hancock R.E.W., Corzo G. (2020). In Vitro and In Vivo Antibiotic Capacity of Two Host Defense Peptides. Antimicrob. Agents Chemother..

[15] Shane A.L., Mody R.K., Crump J.A., Tarr P.I., Steiner T.S., Kotloff K., Langley J.M., Wanke C., Warren C.A., Cheng A.C. (2017). 2017 Infectious Diseases Society of America Clinical Practice Guidelines for the Diagnosis and Management of Infectious Diarrhea. Clin. Infect. Dis..

[16] Veeraraghavan B., Pragasam A.K., Bakthavatchalam Y.D., Ralph R. (2018). Typhoid fever: Issues in laboratory detection, treatment options & concerns in management in developing countries. Future Sci. OA.

[17] Haney E.F., Straus S.K., Hancock R.E.W. (2019). Reassessing the Host Defense Peptide Landscape. Front. Chem..

[18] Fusco A., Savio V., Cammarota M., Alfano A., Schiraldi C., Donnarumma G. (2017). Beta-Defensin-2 and Beta-Defensin-3 Reduce Intestinal Damage Caused by *Salmonella* Typhimurium Modulating the Expression of Cytokines and Enhancing the Probiotic Activity of *Enterococcus faecium*. J. Immunol. Res..

[19] Mookherjee N., Anderson M.A., Haagsman H.P., Davidson D.J. (2020). Antimicrobial host defense peptides: Functions and clinical potential. Nat. Rev. Drug Discov..

[20] Yacoub T., Rima M., Karam M., Fajloun J.-M.S.A.Z. (2020). Antimicrobials from Venomous Animals: An Overview. Molecules.

[21] Arenas I., Villegas E., Walls O., Barrios H., Rodríguez R., Corzo G. (2016). Antimicrobial Activity and Stability of Short and Long Based Arachnid Synthetic Peptides in the Presence of Commercial Antibiotics. Molecules.

[22] Vasilchenko A.S., Rogozhin E.A. (2019). Sub-inhibitory Effects of Antimicrobial Peptides. Front. Microbiol..

[23] Lei J., Sun L., Huang S., Zhu C., Li P., He J., Mackey V., Coy D.H., He Q. (2019). The antimicrobial peptides and their potential clinical applications. Am J Transl Res..

[24] Ji S.Y., Lee H., Hwangbo H., Hong S.-H., Cha H.-J., Park C., Kim D.-H., Kim G.-Y., Kim S., Kim H.-S. (2020). A Novel Peptide Oligomer of Bacitracin Induces M1 Macrophage Polarization by Facilitating Ca(^2+^) Influx. Nutrients.

[25] Payoungkiattikun W., Joompang A., Thongchot S., Nowichai B., Jangpromma N., Klaynongsruang S. (2020). Evidence of multi-functional peptide activity: Potential role of KT2 and RT2 for anti-inflammatory, anti-oxidative stress, and anti-apoptosis properties. Appl. Biol. Chem..

[26] Małaczewska J., Kaczorek-Łukowska E., Wójcik R., Rękawek W., Siwicki A.K. (2019). In vitro immunomodulatory effect of nisin on porcine leucocytes. J. Anim. Physiol. Anim. Nutr..

[27] Scott M.G., Dullaghan E., Mookherjee N., Glavas N., Waldbrook M., Thompson A., Wang A., Lee K., Doria S., Hamill P. (2007). An anti-infective peptide that selectively modulates the innate immune response. Nat. Biotechnol..

[28] Dupont A., Kaconis Y., Yang I., Albers T., Woltemate S., Heinbockel L., Anderson M., Suerbaum S., Brandenburg K., Hornef M.W. (2015). Intestinal mucus affinity and biological activity of an orally administered antibacterial and anti-inflammatory peptide. Gut.

[29] Marin M., Holani R., Blyth G.A.D., Drouin D., Odeón A., Cobo E.R. (2019). Human cathelicidin improves colonic epithelial defenses against *Salmonella* Typhimurium by modulating bacterial invasion, TLR4 and pro-inflammatory cytokines. Cell Tissue Res..

[30] Wuerth K.C., Falsafi R., Hancock R.E.W. (2017). Synthetic host defense peptide IDR-1002 reduces inflammation in *Pseudomonas aeruginosa* lung infection. PLoS ONE.

[31] Marques-Neto L.M., Trentini M.M., das Neves R.C., Resende D.P., Procopio V.O., da Costa A.C., Kipnis A., Mortari M.R., Schwartz E.F., Junqueira-Kipnis A.P. (2018). Antimicrobial and Chemotactic Activity of Scorpion-Derived Peptide, ToAP2, against *Mycobacterium massiliensis*. Toxins.

[32] Bacalum M., Radu M. (2015). Cationic Antimicrobial Peptides Cytotoxicity on Mammalian Cells: An Analysis Using Therapeutic Index Integrative Concept. Int. J. Pept. Res. Ther..

[33] Wu S., Zhang F., Huang Z., Liu H., Xie C., Zhang J., Thacker P.A., Qiao S. (2012). Effects of the antimicrobial peptide cecropin AD on performance and intestinal health in weaned piglets challenged with *Escherichia coli*. Peptides.

[34] Machado R.J.A., Estrela A.B., Nascimento A.K.L., Melo M.M.A., Torres-Rêgo M., Lima E.O., Rocha H.A.O., Carvalho E., Silva-Junior A.A., Fernandes-Pedrosa M.F. (2016). Characterization of TistH, a multifunctional peptide from the scorpion *Tityus stigmurus*: Structure, cytotoxicity and antimicrobial activity. Toxicon.

[35] (2011). Guide for the Care and Use of Laboratory Animals.

[36] de Aluja A.S. (2002). Laboratory animals and official Mexican norms (NOM-062-ZOO-1999). Gac. Med. Mex..

[37] Noster J., Chao T.C., Sander N., Schulte M., Reuter T., Hansmeier N., Hensel M. (2019). Proteomics of intracellular Salmonella enterica reveals roles of Salmonella pathogenicity island 2 in metabolism and antioxidant defense. PLoS Pathog..

